# Factors Influencing the Extent of the Commercialization of Indigenous Crops Among Smallholder Farmers in the Limpopo and Mpumalanga Provinces of South Africa

**DOI:** 10.3389/fsufs.2021.777790

**Published:** 2022-01-25

**Authors:** Nonkululeko Thandeka Brightness Zondi, Mjabuliseni Simon Cloapas Ngidi, Temitope Oluwaseun Ojo, Simphiwe Innocentia Hlatshwayo

**Affiliations:** 1 Centre for Transformative Agricultural and Food Systems, School of Agricultural, Earth and Environmental Sciences, College of Agriculture, Engineering and Science, University of KwaZulu-Natal, Pietermaritzburg, South Africa; 2 African Centre for Food Security, School of Agricultural, Earth and Environmental Sciences, College of Agriculture, Engineering and Science, University of KwaZulu-Natal, Pietermaritzburg, South Africa; 3 Department of Agricultural Extension and Rural Resource Management, School of Agricultural, Earth and Environmental Sciences, College of Agriculture, Engineering and Science, University of KwaZulu-Natal, Pietermaritzburg, South Africa; 4 Department of Agricultural Economics, Obafemi Awolowo University, Ile-Ife, Nigeria; 5 Disaster Management Training and Education Centre for Africa, University of the Free State, Bloemfontein, South Africa

**Keywords:** smallholder farmers, indigenous crops, household commercialization index, double-hurdle fractional response model, South Africa

## Abstract

Smallholder farmers encounter countless challenges that not only restrict them from maximizing market opportunities but also limit their access to the markets. This paper aims to achieve a thorough understanding of the factors that influence the market participation of indigenous crops by smallholder farmers while also analyzing the extent of market participation in South Africa. An analyzable sample size of 1,520 was used for the study. Household commercialization index (HCI), *T*-test, description analysis, and a double hurdle model with quasi-maximum likelihood fractional response model were employed to analyze the commercialization and extent of commercialization among indigenous crops by smallholder farmers in South Africa. The study demonstrated that a farmer’s decision to participate in the market is highly dependent on gender, off-farm income, access to market information, and a family member being infected by HIV. Factors such as household size and access to the market had statistical significance in the extent of market participation by smallholder farmers. While we recommend the need to intensify appropriate training for farmers and extension workers involved in the area of indigenous crops, it is also important that indigenous crops are given the necessary considerations by the government and research institutions so that their demand in the market could increase. There is a need to develop a clear support plan for the few farmers that have decided to be involved in the farming of indigenous crops even though they are not highly marketable. On the other hand, there is also a need for consumer awareness campaigns in South Africa, on the income and nutritional benefits of indigenous crops.

## Introduction

The agricultural sector is the backbone of many South African households (Pawlak and Kołodziejczak, 2020). The majority of households residing in rural areas not only depend on agriculture for their livelihoods and well-being but also are involved in subsistence agriculture which is characterized by a combination of crop and animal production (Shackleton et al., 2001; Gautam and Andersen, 2016). As reported by Statistics South Africa (2019) in the General Household Survey report, about 15.3% of the households in South Africa are involved in agriculture. Some of the crops being produced by these households are ecologically resilient indigenous crops that can withstand a changing climate and other challenges (Akinola et al., 2020). “Indigenous crops are defined as plant species that are either genuinely native to a particular region, or which were introduced to that region for long enough to have evolved through natural processes or farmer selection” (Mahlangu, 2014). These crops are not only a source of income for several smallholder farmers but also the primary source of nutrients for food and nutritional security. Furthermore, they can contribute to sustainable food systems under climate change since they are ecologically resilient (Akinola et al., 2020; Hlatshwayo et al., 2021). Gitz et al. (2017) reported that about 70% of the poor residing in rural areas, completely or partially depend on indigenous crops for their livelihoods. However, smallholder farmers especially in developing countries are faced with numerous constraints that hinder their commercialization of indigenous crops (Meemken and Bellemare, 2020).

Smallholder farmers are faced with a lack of production and marketing knowledge, information, and skills that could enable them to compete with commercial farmers (Minot and Sawyer, 2016). Moreover, the majority of smallholder farmers are also faced with poor quality infrastructure, inadequate storage facilities and transport, and are associated with a lack of information and skills which leads to high transaction costs of participating in the market and hence, over-reliance on traditional social networks and mechanisms for marketing their produce (Ali et al., 2021). Some smallholder farmers still use bartering or gifts to exchange or obtain seeds and crops.

The literature also revealed that other factors that affect the commercialization of indigenous crops are socio-demographic factors such as education level, market information access, HIV status, source of income, and age (Key et al., 2000; Adenegan and Adewusi, 2007; Lubungu et al., 2012; Dlamini-Mazibuko et al., 2019). Smallholder farmers in South Africa are generally illiterate, aged and lack market information (Hlatshwayo et al., 2021). Smallholder farmers operate on small pieces of land which leaves them with no choice but to consume most of their produce with a minimum to sell (Rapsomanikis, 2015). Lack of land possession limits farmers from engaging in long-term investment and makes it difficult for them to access credit. Also, the government and policymakers do not recognize them and hence the benefits of indigenous crops remain unknown (Von Loeper et al., 2016).

While some studies (Karaan et al., 2006; Akinnifesi et al., 2007; Akinola et al., 2020) have been conducted in Africa on several agricultural experiments with regards to indigenous crops and their economic potential, South Africa does not have much information on the factors that affect the participation of smallholder farmers in the commercialization of indigenous crops. Mahlangu (2014) reported that there is a market for indigenous crops and, therefore, it is important to understand the impact of market participation on the livelihoods of smallholder farmers in South Africa. Lack of knowledge on the impact of marketing the indigenous crops could be the reason why some smallholder farmers are not participating in the market in the first place. The provision of information on the benefits of commercializing these crops together with the strategies to address the factors that affect their market participation will be useful to smallholder farmers and may not only influence them but also open up opportunities for them to grow for commercial purposes. Understanding the contribution of indigenous crops toward rural households or smallholder farmers’ livelihoods and food security can raise awareness with regard to effective participation. They can adopt strategies to grow these and sell them to the market. The information will be useful in the implementation and formulation of relevant strategies to commercialize indigenous crops effectively.

The main purpose of this study was to identify the factors that influence the commercialization and the extent of the commercialization of indigenous crops among smallholder farmers since very little is known about the issue. It was clear from the literature that some farmers had been suffering from a lack of market information, farming equipment, and support from extension services. This should be addressed as it results in farmers making poor decisions about which channels of the market to participate in. Lastly, in line with its objective, this study has provided recommendations that will help the government with information about the importance of allowing smallholders to participate in the market, and policymakers to recognize the role of smallholder farmers in market participation.

## Research Methodology

### The Study Area Description

This study was conducted in the Northern and North-East Regions of South Africa covering about two of the nine provinces in South Africa. Limpopo and Mpumalanga provinces are populated by smallholder communal farmers that mainly depend on agricultural and livestock farming for their livelihoods. Limpopo is situated in the Northern part of South Africa, covering about 125,754 km^2^ of the area, which is only 10.2% of the total area of the country. Its population is about 5,8 million with five districts of Mopani, Vhembe, Capricorn, Waterberg and Sekhukhune (Christopher, 2017). The people in this province are highly involved and dependent on agriculture for survival, as 89% of the peoples’ occupation is agriculture. The study was conducted in the districts mentioned above.

The second study area is Mpumalanga province, which is located in the North-Eastern part of South Africa. It covers about 6.5% of the country’s land area. It consists of about 4.04 million people with 72% being involved in agriculture (Christopher, 2017). The overall rainfall received in this province per year is about 1,000 mm, with its warm and temperate weather conditions as it lies 665m above sea level. This province contributes to the agricultural economy through farms produce such as corn (maize), sugar, cotton, groundnuts, potatoes, wheat, and indigenous crops such as Amaranth, Vegetable Cow-pea, African eggplant, Okra and pumpkin (Lehohla, 2016). A variety of fruit is also produced in this province including mangoes and oranges in the subtropical low-veld, whereas peaches are produced at higher elevations.

### Data Types, Sources and Methods of Data Collection

While the data analyzed in this study focuses on two provinces, the research was part of a bigger baseline assessment study that was conducted in four provinces in South Africa. Therefore, the data used in this study has been extracted from an assessment study that was conducted in various provinces of South Africa by the South African Vulnerability Assessment Committee (SAVAC), led by the Secretariat hosted in the Department of Agriculture, Land Reform and Rural Development (DALRRD) in 2016. Data collected included the demographics of the participants, crops (indigenous and cash crops) produced and consumed by rural households, food security and nutrition information. However, the purpose of this particular study is to assess the factors influencing the extent of indigenous crops’ smallholder farmers’ market participation. Smallholder farmers were asked to list the different types of crops they produce, consume and sell. From the list of crops identified by the smallholder farmers, indigenous crops were selected (Table 1).

The study used a quantitative research method to collect data. The multi-stage stratified random sampling technique was used to select households’ representatives’ samples. Characteristics such as institutional factors, sales, socio-economic characteristics, household sizes, and outputs were used to divide the farmers into groups in each site. The DAFF surveys covered random samples of about 4,286 rural smallholder farmers in four provinces of the country. However, this study has only focused on two of the provinces, namely Mpumalanga and Limpopo, with a total of 1,520 respondents selected.

### Methods of Data Analysis

The most used econometric analytical techniques in the analysis of crop market participation include Tobit models, double-hurdle models, and Heckman sample selection models (Donkor et al., 2018; Chen et al., 2020; Tafesse et al., 2020). The Heckman approach is more suitable for incidental truncation where the unobserved values are represented by the zeros; for instance, in situations of wage rate models where the unemployed people are included in the sample (Heckman, 1976; Cameron and Trivedi, 2005). This study used the double-hurdle model, which has been used by many previous studies to analyze the determinants of market participation (Reyes et al., 2012; Achandi and Mujawamariya, 2016; Ingabire et al., 2017).

The double-hurdle model is a two-step decision model: (1) the household decides whether or not to participate in the indigenous crop market and (2) the household decides on the volume of indigenous crops to be marketed. The double-hurdle model (DHM) is known to be a corner solution outcome in dealing with issues of agricultural commercialization of smallholder farmers. This model allows for two types of zero: always zeros, and corner solutions (so that participants have to overcome two hurdles instead of one to sell a positive quantity). It was perfect for this study because it estimates unbiased, efficient and consistent parameters following numerous studies that have applied it (Komarek, 2010; Mather et al., 2013; Achandi and Mujawamariya, 2016). Also, it does not require the participation decision and the participation intensity to be determined in the same process. In the first stage of the double-hurdle model, values of 1 and 0 are assigned to represent the choice of the smallholder farmer’s decision on whether to commercialize the produce or not. Then in the second stage, the factors that determine the extent of commercialization of indigenous crops sold to the market were analyzed using a fractional response model with quasi-maximum likelihood.

A smallholder farmer’s decision to participate in indigenous crops marketing can be represented by:

(1) $$\;Market\_Part_{i}^{*}=x_{i}\beta +e_{i}$$

Where $Market\_Part_{i}^{*}\;$ is the latent variable that indicates whether or not the farmer participates in the market (sells the crops), *x* is a vector of observed independent covariates explaining the decision of market participation, *β* is an unobserved parameter that is to be estimated and *e*_*i*_ is an unobserved error term capturing all other factors, and *e*_*i*_*N*(0, 1).

(2) $$MARKET_{-}PART_{i}=\left\{\begin{array}{l} 1\;if\;Y_{i}^{*}>0\\ 0\;if\;Y_{i}^{*}<0 \end{array}\right\}$$

*MARKET*_*PART*_*i*_ is positive *MARKET*_*PART*_*i*_ = 1 if a farmer effectively participates in the selling of crop (as a seller), i.e. $Market\_Part_{i}^{*}>0$, and *MARKET*_*PART*_*i*_ = 0 or negative if a farmer *i* chooses not to or does not sell in the market. $Y_{i}^{*}$ is the quantity of crops sold by smallholder farmer *i* Conditional to market participation decision.

In accurate terms, the probit model in stage one of assessment is expressed as:

(3) $$\text{Pr}(MARKET\_PART_{i})=X_{0}+X_{1}\beta_{1}+X_{3}\beta_{3}\ldots X_{n}\beta_{n}+e$$

Where Pr (*MARKET*_*PART*_*i*_) is a smallholder farmer’s probability of settling to participate in the market in the form of selling their produce or not, *X*_0_ is a constant parameter, whereas *X*_1_ … *X*_*n*_ are parameters are to be estimated, *β*_1…_*β*_*n*_ are identified in Tables 1 and 2 respectively as the vector of explanatory variables, *e* represents an error term.

From the probit model of the first hurdle, the Inverse Mills Ratio (IMR) is predicted and included as a regressor in the second stage (second hurdle). This is done purposefully to control the selection bias to obtain unbiased, consistent as well as efficient estimators using ordinary least squares. The IMR equation is expressed as follows:

(4) $$\frac{\phi \left[v\left(\frac{P_{i}}{\alpha }\right)\right]}{\phi \left(P_{i}\alpha \right)}$$

Where *ϕ* denotes the normal probability density function. Following is the second-stage equation:

(5) $$E=Y/Z=f\left(x_{i}\beta_{i}\right)+e\frac{\phi \left[v\left(\frac{P_{i}}{\alpha }\right)\right]}{\phi \left(P_{i}\alpha \right)}$$

Where *E* denotes the assumption operator, *Y* representing the (continuous) extent of the vegetables sold in the market, *x* influences the volume of the vegetables sold and is known as the vector of independent variables, and *β* is said to be the vector of the comparing coefficients to be assessed.

In the second hurdle, the study employs the method recommended by Papke and Wooldridge (1996). A fractional response model was employed to estimate the level of commercialization while taking into account the type of dependent variable. The generated sample selection term IMR from the probit model (first hurdle) was fitted as an exogenous variable to account for potential selectivity bias in the fractional response model regarding the level of commercialization (Wooldridge, 2012). The second stage (level of commercialization) equation is expressed as:

(6) $$E\left(HCI_{i}/MARKET_{-}PART_{i}=1\right)=f\left(P_{i},\beta \right)+\omega \lambda$$

(7) $$E\left(HCI/X_{i}HCI>0\right)=\alpha \left(X_{i}\text{Ψ}\right)+\omega \lambda$$

where *HCI* is the observed response on the level of commercialization (HCI)^1^, *E* is the expectation operator; *P*_*i*_ is a vector of the household characteristics; *β* is a vector of parameters to be estimated; λ is the IMR which accounts for sample selection bias in the probit model, and *ω* is the associated parameter to be estimated.

Following Cardoso et al. (2010), assumptions of independence and dominance of the double-hurdle model entered multiplicatively into the log-likelihood function. This allows the two parts of the DH model to be estimated separately: the participation process by a probit regression model while the second hurdle was estimated with the aid of fractional logit QML estimation approach on the sub-sample of positive observations of HCI with the Inverse Mills Ratio used as a regressor in the estimation for correcting selection bias.

The Household Commercialization Index (HCI) was useful in the analysis of the level of indigenous crop output marketed by the smallholder farmers. This is a tool that is used to determine the specific level of commercialization that each household contributes to the market. The most frequently used method of measuring agricultural commercialization in the literature is the proportion of the value of crop sold concerning the value of crop harvested (Chukwukere et al., 2012; Ochieng et al., 2016; Nwafor and van der Westhuizen, 2020). The index can be expressed as follows:

(8) $$HCI_{i}=\frac{\;Gross\;value\;of\;crop\;sales\;hhi\;year\;j}{\;Gross\;value\;of\;all\;crop\;production\;\text{hh}i\;year\;j}\times 100$$

The index measures the ratio of the gross value of indigenous crop sales by household *i* in year j to the gross value of all indigenous crops produced by the same household *i* in the same year *j* expressed as a percentage. The index measures the extent to which household indigenous crop production is oriented toward the market. Thus, a value of zero would be an indication of a subsistence-oriented smallholder farmer whereas the closer the index is to 100, the higher the degree that the smallholder farmer is market-orientated (Hailua et al., 2015). The advantage of this approach is that commercialization is treated as a continuum thereby avoiding a crude distinction between “commercialized” and “non-commercialized” households.

## Results

### Demographic and Socioeconomic Characteristics of the Household Involved in the Production and Marketing of Indigenous Crops

This sub-section of this study presents the demographic and socioeconomic features of the respondents, from a sample of 1,520 rural households. The study found these features to be of great assistance in the matter of distinctly portraying the respondents’ diverse backgrounds and the impact diversity has had on the descriptive, statistical, and econometric results. The results revealed that out of 1,520 smallholder farmers that participated in the study, about 209 were involved in the production of the indigenous crops. Out of 209 smallholder indigenous farmers, about 41 were involved in the commercialization of the identified indigenous crops (Table 1). As presented in Table 3, the mostly produced indigenous crop was pumpkin (36%), followed by leafy vegetables (16%). The least (0.5%) produced indigenous crop was sorghum. This could be as a result of sorghum not being popularly consumed as food but mainly used as an ingredient in the production of traditional beer.

Table 4 showed the different indigenous crops sold by smallholder farmers. As represented in Table 4, the results showed that leafy vegetables were the most sold indigenous crops compared to the other crops. It was also revealed that indigenous crops such as millet, eggplant and cowpea were not commercialized. A possible explanation for this could be the fact that the crops were not enough for both consumption and selling. While pumpkin was found to be the most grown indigenous crop, the extent of its commercialization was limited, suggesting the need to encourage smallholder indigenous crop farmers to participate in the market.

The result of the *t*-test as shown in Table 5, revealed that the smallholder indigenous crop farmers’ mean age and education were not significantly different among the market participants and non-participants. As represented, the mean age for smallholder farmers that participated in the market was 47.33 years, whereas the ones that did not participate in the market had a mean age of 44.23 years. Furthermore, the mean number of years of education for market participants was 9.16 as compared to the non-participants with an average of 5.44 years of formal education. This implies that the literate farmers could at least read, write and hold conversations about commercial farming. Also, they had greater opportunities in terms of taking their farming ventures into other levels of success, including having more international market access than the illiterate ones. The mean output of indigenous crops together with market participation had a significant difference (*P* < 0.05). From the results, at least 800.69 kg was the average yield that was harvested specifically for market participation, whereas only 200.17 kg represented those that did not participate in the market. The higher market yield for participant farmers meant that they had the privilege of consuming and selling at the same time. Amongst all the other tables of this study with demographic characteristics of smallholder farmers in Limpopo and Mpumalanga, South Africa, Tables 5, 6 present various means and standard deviations.

### The Distributions of Commercialization Level of Indigenous Crops

**Figure 1:**
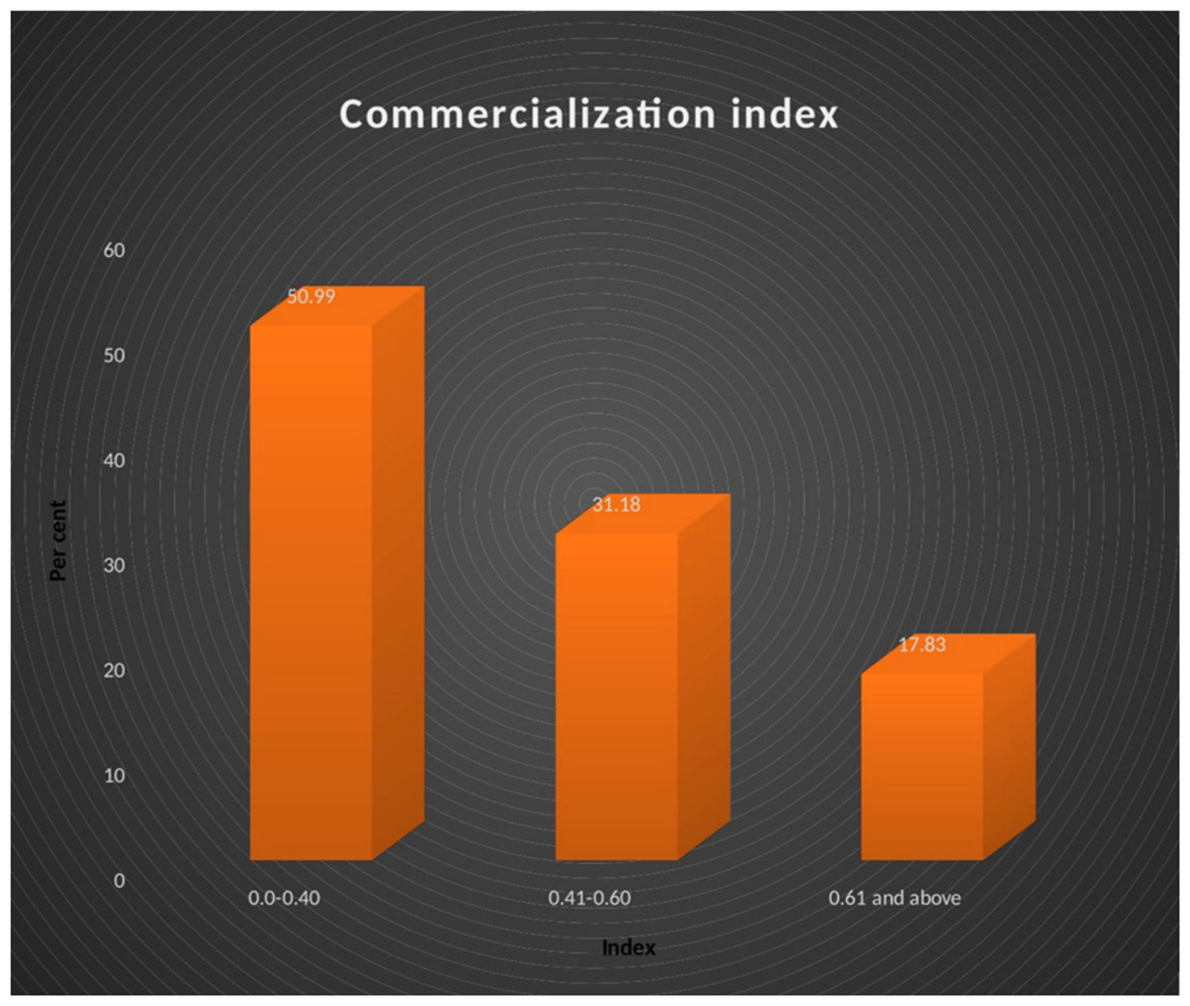
Distribution of the commercialization index of indigenous crops.

According to Strasberg et al. (1999) the household commercialization index (HCI) measures the extent to which indigenous vegetable production gravitates toward the market. The index indicates variations in the level of indigenous vegetable commercialization across the study area. Households were divided into three categories of equal size according to their HCI. As shown in Figure 1, different levels of indigenous crops commercialization among the farmers in the study area are revealed. The results of Figure 1 show that half of the farmers (51%) are still operating almost at the subsistence level. In the same vein, a large number (31%) of the farmers are still at low-medium levels while the rest (18%) are high-level market participants.

While estimating the double-hurdle with the Quasi-maximum likelihood fractional response model, the covariates included in the models were tested for multi-collinearity using the variance inflation factor (VIF). An average VIF of 2.17 shows that the multi-collinearity problem is not an issue among the covariates used for the study. The probit model (first hurdle) was used to estimate factors influencing the commercialization of indigenous crops among the respondents in the study area. The results are shown in Table 7, where the first-hurdle model of the double-model revealed that only the salary of the household and agricultural information was significant at the 1% level. Surprisingly, education had not only no significant impact to the smallholder farmers commercializing their produce, but also it had an unexpected negative coefficient.

From the Probit results, the gender of the household head had a positive effect, at a 5% level of significance, on the determinants of the commercialization of indigenous crops. The results showed that off-farm income had a positive impact, and statistically significant at the level of 5%. Furthermore, the results revealed that if a household member was HIV positive that had a negative effect on the market participation of the farmer with a 10% level of significance. The results show that a household with a member infected with HIV is likely not to participate in the market.

### The Determinants of the Commercialization Level of Indigenous Vegetable Production: A Quasi-Maximum Likelihood Estimates Fractional Logit Model

The results on factors influencing the level of commercialization among indigenous vegetable farmers are as presented in Table 8. The variable household size, gender, marital status, access to information, access to extension, and disability grant were statistically significant and discussed. To correct for selectivity bias, an inverse mill ratio (IMR) was used as a covariate in the model (second hurdle). The IMR was not statistically significant which shows that bias due to selection was not a problem. Hence, using a double hurdle model for estimating determinants and level of commercialization is justified. From the second-hurdle equation it was revealed that the household size, gender of the household head, marital status, access to extension as well as the disability grant were all statistically significant.

The results show that the household size had a positive influence on the level of participation of smallholder farmers with statistical significance of level 10%. Contrary to the first-hurdle model, the coefficient of gender of the household head was negative and statistical significant in influencing market participation. The results also revealed that both marital status and access to information were statistically significant at level 5%, even though their coefficients were opposite (marital status had a positive coefficient whereas access to information had a negative coefficient). It was also revealed that the number of indigenous crops sold in the market was positively influenced by the access to extension services, with a 1% of significance level. This study showed that the disability grant had a negative impact on the level of participation to the market by smallholder farmers with the significance level of 10%.

## Discussion

The objective of the study was to determine the factors influencing the extent of commercialization of indigenous crops among smallholder farmers.

### The Factors That Influence the Smallholder Farmers’ Decision to Participate in the Market

The positive coefficient sign in the gender of the household head implies that gender plays a huge role in the commercialization of indigenous crops. It also provides a clear implication that when men and women work together, they achieve a positive outcome. Sebatta et al. (2014) asserted that men hold the responsibility of deciding whether to participate in the market or not and how much. On the other hand, women become more active in the marketing of arable crops. However, the results of this study differ from what Hill and Vigneri (2014) found in their study as they reported that men practice cash crops farming for the sake of taking care of their families, whereas women produce crops mainly for consumption purposes. This implies that they do not work together in the production of indigenous crops.

Gaining access to information is a key factor that mostly influences farmers’ decisions to participate in the marketing of a product. Farmers with access to information can make informed decisions concerning production, crops to grow, and marketing-related information (Dlamini-Mazibuko et al., 2019). Access to information offers farmers the opportunity to make proper decisions relative to favorable product prices and transaction costs (Key et al., 2000). Having access to market information plays a huge role in the decision-making process of the farmers, about how much to sell and on which market. Farmers require such information to make the appropriate decision on the quantity of produce to market and the price to charge and also to have an idea of the market competition. As revealed from the result of this study, access to marketing information has a positive and statistically significant influence on commercialization among indigenous vegetable farmers. The result of this study aligns with the study of Dlamini-Mazibuko et al. (2019) who, in their study on factors affecting the choice of marketing outlet selection strategies by smallholder farmers in Swaziland, found a positive influence of market information, on the decision to participate in the market.

The positive coefficient implies that the involvement of indigenous crop farmers in off-farm income opportunities increases their likelihood of commercializing indigenous crops. This implies that regardless of households’ other means of making income, they still manage to produce crops and participate in the market. Usually, households that have other means of making income are business-minded and can multitask. Other than farming, females always get involved in other streams of generating income, especially in female-headed households. They partake in casual jobs to take care of their families’ needs. The results of this study corroborate that of Mthembu (2013) who from their findings showed that women were not only involved in the production and marketing of indigenous crops, but also worked as road maintainers, with others sewing and making grass mats.

The negative coefficient result indicates that a household with a member infected with HIV is likely not to participate in the market. This could be attributed to the fact that farmers tend to spend more time taking care of the sick and less time trying to make produce. From the study conducted by Khapayi and Celliers (2016), having a sick member could reduce labor which negatively impacts the decision to participate in the market because smallholder production depends highly on family labor in the production of crops. This result is consistent with Adenegan and Adewusi (2007) who concluded that if the number of HIV-infected households increases in the rural areas then their survival strategies together with food security get threatened as well.

### The Determinants of the Level of Market Participation of Smallholder Farmers

The coefficient of access to extension had a statistically positive influence on the household level of commercialization. This study aligns with the study of Ojo and Baiyegunhi (2020) who found a positive relationship between access to extension service and the farmers’ choice of the adoption of a climate change adaptation strategy. Extension services improve the understanding of farmers, which leads to higher production, a higher probability of participating in the market, and the commercialization of indigenous crops. The results of this study comply with the study of Martey et al. (2012) who indicate that the extent of commercialization is determined by farmers’ access to extension service. Therefore, the importance of providing timely access to extension services would significantly contribute to how farmers make their decisions when planning to commercialize the production and marketing of indigenous crops.

The positive coefficient illustrates that as the number of household members increases, so does the quantity sold in the market. These results are in contrast with what Kyaw et al. (2018) reported. In their findings, they discovered that the size of the household had a negative impact and it was statistically significant. It was then concluded that large households are associated with fewer agricultural products to sell in the market due to prioritizing the consumption needs of the household. The findings were substantiated by Siziba et al. (2011) who posited that the larger the number of households in the family the lesser the chances of them marketing the quantity that is beyond their consumption satisfaction.

The coefficient of gender of household head was negatively signed and statistically significant. It was no surprise that the results came out negative, and this is mainly because female-headed households are more likely to be involved in the market of indigenous crops as compared to the male counterparts who mostly involved in the harvesting and marketing of cash crops. The result is in tandem with other studies conducted by Shackleton and Shackleton (2006) and Avocèvou-Ayisso et al. (2009) in South Africa and Nigeria, respectively. This study found the gender of the household head to be a significant determinant in marketing of indigenous crops. In the same vein, the findings of Sinyolo et al. (2017) revealed that female-headed households sold more quantities of maize than male-headed households. However, the results of this study were in variance with those of the past numerous studies (Boughton et al., 2007; Hlongwane et al., 2014; Sigei et al., 2014) who posited that female-headed households not only lack extension services but also suffer from limited information access on trends and the inability to secure greater contracts that could help provide them with better markets for their crops.

Contrary to the first hurdle, access to market information indicated a negative impact on the volume of the indigenous crops to be sold in the market. The result shows that the more the indigenous crops farmers has access to the market information, the volume offered for sale reduces. This is unexpected from the economic point of view. However, the plausible reason could be attributed to inadequate or unqualified staff members and poor organization, which could limit the efficient dissemination of market information (De et al., 2005; Ojo et al., 2019; Bello et al., 2020). As a result, market information might not be disseminated as efficiently as expected. This could be the fact that most indigenous crop farmers reside in remote areas with a lack of good quality infrastructure, skills in agricultural activities, and inadequate storage facilities and transport (Minot and Sawyer, 2016). This not only impedes their opportunity for competing in the international markets but also deprives them of the opportunities of earning a living through the commercialization of indigenous crops. Also, this infers that being unable to sell their produce, smallholder farmers could end up not partaking in the agricultural sector at all.

The positive relationship between the access to extension services and farmer’s commercialization of indigenous crops implies that having access to extension services provides the smallholder farmers with the privilege of being aware of market availability. It also comes in handy in enhancing their knowledge of production by making them aware of information on improved varieties. These results manifest the importance of the urgency of implementing not only improved technology but also support services in the promotion of market sales. Apind et al. (2015) found similar positive results to this study specifying that the extension services had a positive coefficient and significantly influenced the volume of rice that the smallholder farmers sold in the market. Alemu et al. (2012) added that the access to extension services in Ethiopia increased the probability of smallholder farmers opting for the contract market rather than settling for the spot market.

The negative coefficient on disability grants implies that the rise in the income of households that receive the disability grant may entrench an entitlement and culture of dependency among them. This could result in them being lazy or even not growing crops, let alone engaging in income-generating ventures. Similar findings were also reported by Aliber and Hall (2012) and Tirivayi et al. (2016). However, not participating in the market could also be due to the physical condition of the smallholder farmer.

## Conclusion And Recommendations

The participation of smallholder farmers in marketing of produce can play a critical role in meeting their goals such as food and nutrition security, poverty alleviation and sustainable agriculture. This study found that the market participation and sales ratio of smallholder indigenous crop farmers are constrained by numerous factors, such as socioeconomic, market and institutional factors. The commercialization of the indigenous crop for smallholder farmers in the market was affected by gender, educational level, off-farm income, agricultural information, and a member being infected by HIV. The household size, gender of household head, access to market information, extension services and disability grant were found to highly influence the extent of commercialization among the smallholder indigenous vegetable farmers.

To fully realize the optimum contribution of indigenous crops to household food and nutrition security, support from the stakeholders must be geared toward the smallholder indigenous farmers through the provision of farm training for an effective and efficient grasp of agricultural and marketing information. To improve smallholder farmers’ access to markets, government also needs to ensure that their support for the production of indigenous crops is timely and well-targeted in order to upscale its production for consumption and commercialization. Where possible, government and other stakeholders need to channel their support through organized cooperatives that exist within the smallholder farmers. Much attention and support need to be given to women’s involvement in market participation, and they also need to be empowered by the government and other interested stakeholders to participate fully in the decision making relating to the price of their produce and where to sell it. More workshops especially for young people and women need to be conducted in rural areas to raise awareness on the nutritional importance of indigenous crops and the need to include these indigenous crops into South Africa’s dietary guidelines.

## Figures and Tables

**Table 1:** Indigenous crops grown by Limpopo and Mpumalanga Smallholder farmers.

Indigenous crops	Scientific names
Amadumbe	*Colocasia esculenta*
Bambara groundnut	*Vigna subterranea*
Cassava	*Manihot esculenta*
Cowpea	*Vigna unguiculata*
Eggplant	*Solanum melongena*
**Leafy vegetables**	
Millet	*Panicum miliaceum*
Okra	*Abelmoschus esculentus*
Pumpkin	*Cucurbita*
Sorghum	*Sorghum bicolor*

*Source: Mabhaudhi et al. (2017)*.

**Table 2:** Estimated factors that affect the smallholder farmers’ decisions for market participation.

Variable name	Variable definition	Variable type and measurement	Market participation effect
Age	Age of the household head	In years (continuous)	±
Gender	Gender of the household head	Dummy (1 = male, 0 = female)	+
Marital status	Marital status of the household head	Marital status (1 = married, 0 = single)	
Household size	Number of members of the household	Size of household (continuous)	−
Educational attainment	Education level of the household head	Education level (continuous)	+
Livestock	Ownership of livestock	Dummy (1 = yes, 0 = no)	±
Distance	Distance to the market	In kilometers (continuous)	−
Credit access	Access to credit	Dummy (1 = yes, 0 = no)	+
Extension services	Access to extension service	Dummy (1 = yes, 0 = no)	+

± *indicates whether the hypothesized effect will be positive or negative*, + *indicate a positive estimated effect, and – indicate the negative estimated effect*.

**Table 3:** Distribution of all the indigenous crops grown by smallholder farmers.

	Indigenous crop farmers in the Mpumalanga and Limpopo provinces	
Indigenous crops	Frequency	Percentage
Sorghum	1	0.5
Pumpkin	75	36
Okra	4	1.9
Millet	3	1.4
Leafy vegetables	63	30
Eggplant	4	1.9
Cowpea	4	1.9
Cassava	4	1.9
Bambara groundnut	15	7
Amadumbe	36	17

**Table 4:** Distribution of all indigenous crops that were sold to the market by smallholder farmers.

	Smallholder indigenous farmers participating in the market	
Indigenous crops	Frequency	Percentage
Sorghum	1	2.4
Pumpkin	8	20
Okra	2	5
Millet	0	0
Leafy vegetables	15	37
Eggplant	0	0
Cowpea	0	0
Cassava	2	5
Bambara groundnut	3	7
Amadumbe	10	24

**Table 5:** Demographic characteristics of smallholder farmers that were involved in the production of indigenous crops in Limpopo and Mpumalanga provinces, South Africa.

Characteristics	Market participation	Mean	*F*-value	Degrees of freedom	*P*-value
Age of the household head	Yes	47.33	1.009	129	0.314
	No	44.23		21.52	
Education of the household head	Yes	9.16	0.000	102	0.989
	No	5.44		17.14	
Total output of the indigenous crops (KG)	Yes	800.69	26.623	318	0.000***
	No	200.17		132.00	

***, **, * *Indicate significance at 1, 5, and 10% level, respectively*.

**Table 6:** Demographic characteristics of indigenous crops’ farmers in Limpopo and Mpumalanga provinces, South Africa.

	Market participants			Non-market participants			Pooled	
Variables	Mean	Standard deviation (SD)		Mean	Standard deviation (SD)		Mean	Standard deviation (SD)
Gender of the household head	0.564	0.112		0.533	0.100		1.27	0.45
Household age (Years)	47.333	13.342		44.443	12.666		49.12	11.89
Marital status	0.465	0.356		0.443	0.344		4.21	2.44
Household size (Numbers)	4.786	1.223		3.889	1.012		4.93	2.71
Educational level of household (Years)	6.678	3.048		5.423	2.345		33.58	40.30
Ownership of livestock	0.587	0.357		0.700	0.327		1.77	0.42
Distance to the market (Km)	0.487	0.356		0.475	0.245		1.86	1.82
Access to market information	0.573	0.785		0.455	0.676		1.94	0.24
Access to agricultural assistance	0.486	0.345		0.428	0.367		1.92	0.27
Family member with HIV	0.444	0.432		0.378	0.421		0.47	0.79
Family member worked on farm	0.655	0.557		0.544	0.447		0.98	0.76
Social grant	0.468	0.367		0.490	0.455		1.99	0.73
Irrigation type	0.586	0.234		0.354	0.345		1.52	0.50
Total output of indigenous crops (kg)	800.69	671.8		200.17.2	6.74		1000.22	768.067

**Table 7:** Probit results for determinants of commercialization of indigenous crops.

Commercialization	Coef.	St.Err.	p-value	dy/dx	Std.Err.	*P*-value
Household size	0.016	0.049	0.740	0.000	0.001	0.740
Gender of the household head	0.849	0.358	0.018**	0.012	0.005	0.018**
Residents of household	−0.092	0.704	0.896	−0.001	0.010	0.896
Educational level	−0.236	0.721	0.744	−0.003	0.011	0.744
Marital status	0.188	1.272	0.882	0.003	0.019	0.883
Agricultural information	2.057	0.564	0.000***	0.030	0.008	0.000***
Involved in livestock prod	−0.511	0.613	0.405	−0.007	0.009	0.404
Off-farm income	1.037	0.418	0.013**	0.015	0.006	0.013**
WEALTHINDEX^a^	1.139	0.260	0.000***	0.017	0.004	0.000***
Access to extension	−0.274	0.334	0.412	−0.004	0.005	0.410
Access to the disability grant	−0.427	1.293	0.741	−0.006	0.019	0.741
Household member with HIV	−1.000	0.542	0.0658*	−0.015	0.008	0.062*
Constant	0.152	0.996	0.878			
Mean dependent var	0.646					
Pseudo r-squared	0.957					
Chi-square	1824.810					
Akaike crit. (AIC)	107.636					
SD dependent var	0.516					
Number of obs	1454.000					
Prob> chi2	0.000					
Bayesian crit. (BIC)	176.303					
VIF	2.17					

a WEALTHINDEX was generated using principal component analysis (PCA) from the list of wealth indicators owned by the indigenous vegetable farmers. The lists are: wall materials of the house, bank account, owning vehicles, owning a TV, owning radio, type of ceiling, the main fuel used for cooking, etc.

^***, **, *^ *Indicate significance at 1, 5, and 10% level, respectively*.

**Table 8:** Determinants of the extent of commercialization of indigenous crops (Quasi-maximum likelihood estimates fractional logit model).

Commercialization index	Coef.	Std.Err.	*P*-value
Household size	0.014	0.008	0.091*
Gender of household head	−0.192	0.108	0.077*
If the household head resident	−0.224	0.215	0.298
Education of household head	0.124	0.226	0.584
Marital status	1.034	0.459	0.024**
Access to information	−0.200	0.094	0.033**
If_HH_involved_in_livestock_prod	−0.107	0.261	0.681
If_member_worked_for_a_wage_salary	−0.206	0.182	0.257
WEATHINDEX	0.168	0.105	0.111
HH_received_advice_from_government	−0.043	0.098	0.659
Access to extension	0.085	0.048	0.078*
Disability grant	−0.888	0.464	0.056*
HIV_if_a_member_has_been_informed	−0.359	0.226	0.112
Inverse mills ratio (IMR)	0.040	0.113	0.724
Constant	0.513	0.406	0.206
Wald chi2(14	30.73		
Prob> chi2	0.0061		
Pseudo R2	0.0060		
Log pseudo-likelihood	−976.31617		

^***, **, *^ *Indicate significance at 1, 5, and 10% level, respectively*.

## Data Availability

The data analyzed in this study is subject to the following licenses/restrictions: This dataset belongs to the South African Vulnerability Assessment Committee (SAVAC), hosted by the Department of Agriculture, Land Reform and Rural Development (DALRRD). If you want it you apply from SAVAC. Requests to access these datasets should be directed to the website for DALRRD, www.dalrrd.gov.za.

## Footnotes

1 The outcome variable for the second hurdle, level of commercialization, is the HCI index from the indigenous farmers in the study area.
